# The correlation between LDH serum levels and clinical outcome in advanced biliary tract cancer patients treated with first line chemotherapy

**DOI:** 10.1038/srep24136

**Published:** 2016-04-11

**Authors:** Luca Faloppi, Michela Del Prete, Andrea Casadei Gardini, Daniele Santini, Nicola Silvestris, Maristella Bianconi, Riccardo Giampieri, Martina Valgiusti, Oronzo Brunetti, Alessandro Bittoni, Kalliopi Andrikou, Eleonora Lai, Alessandra Dessì, Stefano Cascinu, Mario Scartozzi

**Affiliations:** 1 Department of Medical Oncology, AOU “Ospedali Riuniti” - Università Politecnica delle Marche, Ancona, Italy; 2 Medical Oncology, University Hospital, University of Cagliari, Cagliari, Italy; 3 IRCCS, Istituto Scientifico Romagnolo per lo Studio e la cura dei Tumori, Meldola, Italy; 4 Department of Medical Oncology, Campus Bio-Medico University of Rome, Roma, Italy; 5 Medical Oncology Unit, Cancer Institute “Giovanni Paolo II”, Bari, Italy

## Abstract

LDH may represent an indirect marker of neo-angiogenesis and worse prognosis in many tumour types. We assessed the correlation between LDH and clinical outcome for biliary tract cancer (BTC) patients treated with first-line chemotherapy. Overall, 114 advanced BTC patients treated with first-line gemcitabine and cisplatin were included. Patients were divided into two groups (low vs. high LDH), according to pre-treatment LDH values. Patients were also classified according to pre- and post-treatment variation in LDH serum levels (increased vs. decreased). Median progression free survival (PFS) was 5.0 and 2.6 months respectively in patients with low and high pre-treatment LDH levels (p = 0.0042, HR = 0.56, 95% CI: 0.37–0.87). Median overall survival (OS) was 7.7 and 5.6 months (low vs. high LDH) (p = 0.324, HR = 0.81, 95% CI: 0.54–1.24). DCR was 71% vs. 43% (low vs. high LDH) (p = 0.002). In 38 patients with decreased LDH values after treatment, PFS and OS were respectively 6.2 and 12.1 months, whereas in 76 patients with post-treatment increased LDH levels, PFS and OS were respectively 3.0 and 5.1 months (PFS: p = 0.0009; HR = 0.49; 95% IC: 0.33–0.74; OS: p < 0.0001; HR = 0.42; 95% IC: 0.27–0.63). Our data seem to suggest that LDH serum level may predict clinical outcome in BTC patients receiving first-line chemotherapy.

Biliary tract cancer (BTC) is a rare group of tumours including gallbladder carcinomas and cholangiocarcinomas (extrahepatic cholangiocarcinoma, intrahepatic cholangiocarcinoma and Klatskin tumour). In Western Countries, BTC has an incidence of 1–2 cases/100.000^1^.

Patients diagnosed with BTC usually have a dismal prognosis with a median overall as poor as 10–12 months for metastatic or locally advanced tumours^1^. Surgery is the only curative treatment for BTC, but only 10–20% of patients are deemed radically resectable in experienced surgical centers. When radical resection is feasible, the median overall survival approaches 36 months, with a recurrence rate as high as 60% in most series^2^. Even with the use of a standard first-line chemotherapy (i.e. gemcitabine in combination with platinum compounds) results for metastatic BTC patients are globally disappointing with a non-negligible proportion of patients rapidly progressing^3^. Although clinical and biological evidence suggests that BTC may represent a heterogeneous group of tumours, there is a substantial lacking of clinical and molecular factors allowing a reliable patients selection^4^.

Research progresses in the understanding of BTC carcinogenesis have led to the discovery of new potentially useful molecular targets for biologically guided treatment options particularly involving tumour angiogenesis^5^,^6^. Chronic inflammation induces cholangiocytes to produce chemokines and cytokines, with a consequential activation of nitric oxide (NO) or cyclooxygenase-2 (COX2) and damage of the DNA mismatch repair machinery. The DNA damage ultimately leads to cell growth, inhibition of apoptosis and promotion of angiogenesis. The vascular endothelial growth factor (VEGF) is one of the principal pathways involved in cholangiocarcinogenesis, facilitating tumour growth and metastasis^7^. In this view an increased microvessel density has been in fact associated with a worse prognosis (lower 5-year survival rates, higher recurrence rates and increased nodal spread)^8^.

Lactic dehydrogenase (LDH) is a glycolytic enzyme, composed of four polypeptide chains, each one encoded by separate gene (M and H) with a key role in the conversion of pyruvate to lactate under anaerobic conditions. Five isoforms of LDH have been identified as a result of the five different combinations of polypeptide subunits. The biological link between hypoxia, LDH levels and the tumour-driven angiogenesis pathway through the abnormal activation of the Hypoxia Inducible Factor 1 α (HIF1-α) is well established^9^,^10^. Since LDH and pro-angiogenesis factors are regulated by the same HIF1α-driven molecular pathway, high LDH levels are usually concomitantly present with the abnormal activation of the VEGF pathway. It has been in fact demonstrated that high LDH serum levels were associated with tumour overexpression of VEGFA and VEGFR in many tumour types^11^,^12^,^13^,^14^. As a consequence, it has been postulated that LDH levels may represents and indirect marker of activated tumour angiogenesis and worse prognosis.

In our analysis, we assessed the role of LDH serum levels in predicting clinical outcome for biliary tract cancer patients treated with first-line chemotherapy with the aim to individuate a potentially reliable and easy to use marker for patients stratification.

## Patients and Methods

### Patients selection

This study is a retrospective multicentre analysis conducted at five institutions in Italy (Department of Medical Oncology, AOU “Ospedali Riuniti”, Ancona; IRCCS, Istituto Scientifico Romagnolo per lo Studio e la cura dei Tumori, Meldola; Department of Medical Oncology, Campus Bio-Medico University of Rome, Roma; Medical Oncology Unit, Cancer Institute “Giovanni Paolo II”, Bari; Medical Oncology, University Hospital, University of Cagliari, Cagliari).

All consecutive patients with histologically proven BTC receiving a first line chemotherapy with gemcitabine (1200 mg/sqm on days 1 and 8 every 21 days) and cisplatin (75 mg/sqm on days 1 every 21 days) from 2007–2014 were eligible for our analysis. Dose reductions were performed in case of toxicity according to current guidelines. LDH values were collected within one month before the treatment and within 1 months after 3 months of chemotherapy.

Follow-up consisted of physical examination, a complete blood count, carcinoembryonic antigen (CEA) and carbohydrate antigen (Ca19.9) assay, CT/MRI scanning as clinically indicated. Tumour response was evaluated every 12 weeks by clinicians’ assessment and according to the Response Evaluation Criteria in Solid Tumours (RECIST version 1.1). The A.O.U. “Ospedali Riuniti” of Ancona Ethical Committee approved the analysis (214684).

All patients included in the study were treated according to approved guidelines. Informed consent was obtained from all subjects.

### Statistical analysis

Statistical analysis was performed by the MedCalc package (MedCalc^®^ v13.1.2.0).

Mann-Whitney test was used to compare groups of patients for objective response at 3 months (progressive vs. stable or responding disease).

LDH cutoff value for study purposes was determined by receiver operating characteristics curve (ROC) analysis, based on objective response at 3 months.

Patients were divided into two groups (A and B, below and above the cut-off level respectively). Patients were also classified according to pre- and post-treatment variation in LDH serum levels (increased vs. decreased).

The association between categorical variables and disease control rate (DCR) were analyzed by chi-square test. Survival distribution was estimated by the Kaplan-Meier method. Significant differences in probability of relapsing between the strata were evaluated by log-rank test. Cox multiple regression analysis was used to assess the role of variables resulted significant at univariate analysis.

Tested variables included gender (male vs. female), median age (<68 yrs vs. ≥68 yrs) and Eastern Cooperative Oncology Group performance status (ECOG PS: 0–1 vs. ≥2) at first line chemotherapy beginning, primitive tumour site (gallbladder tumours vs. intrahepatic cholangiocarcinoma vs. extrahepatic distal cholangiocarcinoma vs. extrahepatic hilar cholangiocarcinoma), previous surgery (yes vs. no), Ca19.9 serum levels (≤vs. > upper normal rate, UNR). A significant level of 0.05 was chosen to assess the statistical significance.

For statistical analysis overall survival (OS) and progression-free survival (PFS) were defined respectively as the interval between the start of treatment to death or last follow-up visit and as the interval between the start of treatment to clinical progression or death or last follow up visit if not progressed.

## Results

**Figure 1:**
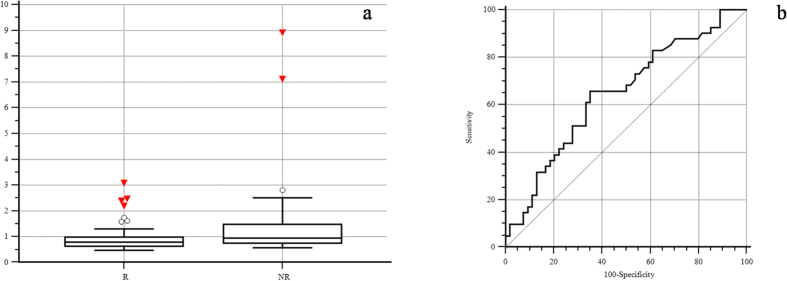
LDH pre-treatment serum levels according to objective response to first line chemotherapy (responders vs. not responders): (**a**) Mann-Whitney test (p = 0.0155); (**b**) ROC curve analysis (p = 0.0112, cut off: ≥0.89).

Globally 114 patients with advanced BTC receiving a first line chemotherapy were available for our analysis. The cut-off point with the highest sensitivity and specificity for estimating pre-treatment LDH serum levels as a function of treatment clinical activity was set at 0.89 times the upper normal range (UNR) after ROC curve analysis (Fig. 1). Consequently patients showing a pre-treatment LDH serum level <0.89 UNR were classified as LDH-low patients (56 patients, 49%, group A) whereas patients with pre-treatment LDH serum level ≥0.89 UNR were classified as LDH-high patients (58 patients, 51%, group B).

The two groups of patients resulted comparable for all major clinical characteristics such as gender, median age, performance status, primitive tumour site, previous surgery (Table 1).

In the whole group, 65 patients showed disease control (disease control rate, DCR 57%) and 49 patients (43%) progressed during the treatment. In patients with high or low pre-treatment LDH serum levels, we observed disease control in 25 (43%) and 40 (71%) cases respectively (p = 0.002). In patients with increased or decreased LDH serum levels after treatment disease control was obtained respectively in 34 (45%) and 31 (82%) cases (p = 0.0001) (Table 1).

**Figure 2:**
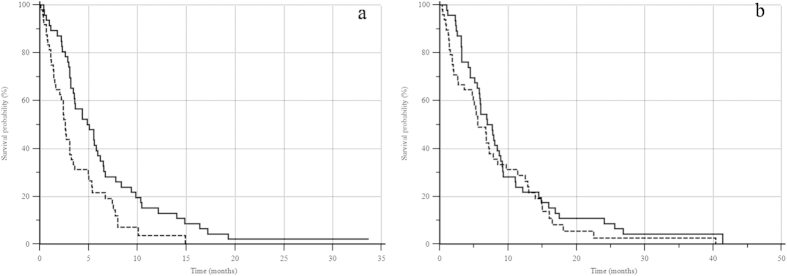
Kaplan-Meier curve analysis of pre-treatment LDH serum levels (low— vs. high -------), according to ROC curve cut-off (≥0.89): (**a**) mPFS: 5.0 vs. 2.6 months (p = 0.0042, HR = 0.56, 95% CI: 0.37–0,87); (**b**) mOS: 7.7 vs. 5.6 months (p = 0.324, HR = 0.81, 95% CI: 0.54–1.24).

Median PFS was 5.0 months and 2.6 months respectively in patients with low and high pre-treatment LDH levels (p = 0.0042, HR = 0.56, 95% CI: 0.37–0.87). Median OS was 7.7 months and 5.6 months (low vs. high LDH) (p = 0.324, HR = 0.81, 95% CI: 0.54–1.24) (Fig. 2).

**Figure 3:**
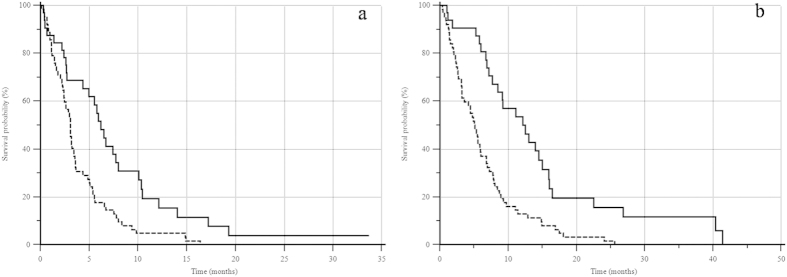
Kaplan-Meier curve analysis according to pre- and post-treatment variation of LDH serum levels (decreased, 35 pts— vs. increased, 79 pts -------): (**a**) mPFS: 6.2 vs. 3.0 months (p = 0.0009; HR = 0.49; 95% IC: 0.33–0.74); (**b**) mOS: 12.1 vs. 5.1 months (p < 0.0001; HR = 0.42; 95% IC: 0.27–0.63).

In the group of 38 patients with decreased LDH levels after treatment, PFS was 6.2 months and OS was 12.1 months, whereas in the 76 patients with increased LDH levels PFS was 3.0 months and OS was 5.1 months (PFS: p = 0.0009; HR = 0.49; 95% IC: 0.33–0.74; OS: p < 0.0001; HR = 0.42; 95% IC: 0.27–0.63) (Fig. 3).

Of all clinical variables tested, ECOG PS and Ca19.9 serum levels were both able to predict PFS and OS. In particular PFS was 4.3 months in case of ECOG PS 0–1 vs 2.4 months in case of ECOG PS ≥2 (p = 0.0015; HR = 0.51; 95% IC: 0.30–0.85). Median overall survival was 7.9 months in case of ECOG PS 0–1 vs. 3.6 months in case of ECOG PS ≥2 (p = 0.0007; HR = 0.48; 95% IC: 0.28–0.82). Median progression free survival was 4.9 months in case of Ca 19.9 serum levels ≤ UNR vs. 2.4 months in case of Ca 19.9 serum levels > UNR (p = 0.0018; HR = 0.53; 95% IC: 0.35–0.81). Median overall survival was 9.3 months in case of Ca 19.9 serum levels ≤ UNR vs. 4.9 months in case of Ca 19.9 serum levels > UNR (p = 0.0001; HR = 0.46; 95% IC: 0.30–0.70).

At multivariate analysis pre-treatment LDH serum level and pre- and post-treatment LDH serum level variation maintained an independent prognostic value for PFS (p < 0.0001). ECOG PS, Ca19.9 serum level, and pre- and post-treatment LDH serum level variation maintained an independent prognostic value for OS (p < 0.0001) (Table 2).

All the other clinical variables analyzed failed to show any correlation with patients outcome.

## Discussion

The lacking of clinical and biological factors able to predict patients’ outcome during first-line chemotherapy is one of the most critical aspect in BTC.

Our analysis showed a statistically significant improved PFS and DCR in patients with low pre-treatment LDH serum levels compared with patients with high pre-treatment LDH levels. We also found statistically significant improved PFS and OS in the group of patients with decreased LDH values after treatment, compared with patients showing increased LDH levels after treatment.

Results about ECOG PS and Ca19.9 serum levels confirm their clinical role in BTC patients.

We believe that the association between high LDH serum levels and tumour angiogenesis may explain the prognostic role of LDH in different solid tumours including BTC^15^,^16^,^17^,^18^,^19^,^20^. Tumour angiogenesis and tumour-induced hypoxia are in fact usually related to poor prognosis and clinical outcome.

In keeping with these considerations Furuse *et al.* suggested a correlation between high LDH levels and poor prognosis in a small analysis of BTC patients receiving uracil-tegafur plus doxorubicin (p = 0.043)^21^. A Further study by Saisho *et al.*, evaluating multiple prognostic factors among 65 patients with advanced BTC receiving chemotherapy, confirmed the possible negative prognostic value of high LDH levels^4^.

Our findings seem to suggest a possible predictive function for LDH serum levels in BTC patients receiving first-line chemotherapy. The correlation with PFS and DCR are suggestive of a predictive activity of LDH and so it is the role of LDH levels variations observed during treatment. Once again the correlation between tumour angiogenesis and LDH levels may represent a potential explanation for these observations. The limited sample size might represent a possible explanation for the lack of independent correlation between LDH serum levels and median overall survival.

Tumor angiogenesis and all related biological phenomena have been in fact suggested to directly influence response/resistance to chemotherapy in different series in particular with the use of platinum compounds^22^,^23^. Accordingly, clonogenic survival of colon cancer cells after oxaliplatin treatment has been shown to improve in hypoxic conditions, whereas chemotherapy-induced DNA adducts were significantly more present in aerobic tumor cells^24^,^25^.

## Conclusions

The possibility to use a widespread, easy to obtain, and potentially reliable marker such as LDH may have a relevant impact in the clinical practice for a better patients’ stratification and selection. The option to further identify different risk-groups in BTC represents in fact a key-challenge for the treatment of this disease, as the risk of toxicity may not be entirely balanced by the presumably poor clinical benefit deriving from first-line chemotherapy in the group of patients with high LDH serum levels. Besides a definite prognostic role, LDH levels variations during chemotherapy may also, at least hypothetically, suggest a treatment strategy re-evaluation, reinforcing or discouraging first-line chemotherapy.

Nonetheless, before these findings can be applied in clinical practice, prospective confirmations are mandatory. Our data may help towards designing future clinical trials with the aim of investigating the outcome of different chemotherapy regimens in different patient groups prospectively stratified according to the prognostic and predictive profile.

## Additional Information

**How to cite this article**: Faloppi, L. *et al.* The correlation between LDH serum levels and clinical outcome in advanced biliary tract cancer patients treated with first line chemotherapy. *Sci. Rep.* **6**, 24136; doi: 10.1038/srep24136 (2016).

## Figures and Tables

**Table 1:** Main clinical characteristics and results for the whole patients population and according to LDH pre-treatment values (above or below the cut-off) and to the LDH variation pre- and post-treatment.

Characteristics	Whole Group	pre-treatment LDH			pre-/post-treatment LDH variation		
		Group A	Group B	p value	LDH	LDH	p value
		*LDH < cutoff*	*LDH ≥ cutoff*		decreased	increased	
	n = 114	n = 56	n = 58		n = 38	n = 76	
Age (range)	68 (31–84)	67 (43–79)	69 (31–84)		67 (43–84)	68 (31–82)	
Gender (%)							
Males	50 (44)	30 (54)	20 (34)	ns	13 (34)	37 (49)	ns
Females	64 (56)	26 (46)	38 (66)		25 (66)	39 (51)	
ECOG PS (%)							
0–1	78 (68)	43 (77)	35 (60)	ns	29 (76)	49 (64)	ns
≥2	36 (32)	13 (23)	23 (40)		9 (24)	27 (36)	
Primitive tumour site (%)							
Gallbladder tumours	25 (22)	11 (20)	14 (24)		8 (21)	17 (22)	
Extrahepatic distal cholangiocarcinoma	17 (15)	7 (12)	10 (17)	ns	6 (16)	11 (15)	ns
Extrahepatic hilar cholangiocarcinoma	11 (10)	8 (14)	3 (5)		4 (10)	7 (9)	
Intrahepatic cholangiocarcinoma	61 (53)	30 (54)	31 (54)		20 (53)	41 (54)	
Previous surgery							
Yes	49 (43)	25 (45)	24 (41)	ns	17 (45)	32 (42)	ns
Not	65 (57)	31 (55)	34 (59)		21 (55)	44 (58)	
Ca19.9 serum levels							
≤upper normal rate	49 (43)	27 (48)	22 (38)	ns	19 (50)	30 (39)	ns
>upper normal rate	65 (57)	29 (52)	36 (62)		19 (50)	46 (61)	
Objective response (%)							
Disease Control (SD + PR)	65 (57)	40 (71)	25 (43)	0.002	31 (82)	34 (45)	0.0001
Not response (PD)	49 (43)	16 (29)	33 (57)		7 (18)	42 (55)	
Survival							
mPFS (months)	3.4	5.0	2.6	0.0042	6.2	3.0	0.0009
mOS (months)	6.8	7.7	5.6	0.324	12.1	5.1	<0.0001

**Table 2:** Results of multivariate analysis for PFS and OS.

Variables	p value	Overall Significance level
PFS		
LDH serum levels pre-treatment	0.0076	<0.0001
LDH serume levels variation pre-/post- treatment	0.0012	
Ca19.9 serum levels	0.1495	
ECOG Performance Status	0.0522	
OS		
LDH serume levels variation pre-/post- treatment	0.0001	<0.0001
Ca19.9 serum levels	0.0185	
ECOG Performance Status	0.0336	
